# The potential of semi-quantitative and quantitative methods in predicting progression in rheumatoid arthritis-associated interstitial lung disease

**DOI:** 10.1007/s10067-025-07443-7

**Published:** 2025-05-15

**Authors:** Duygu Temiz Karadag, Sevtap Dogan, Ozgur Cakir, Yusuf Altıntas, Seyma Yilmaz, Neslihan Gökcen, Ayten Yazici, Ayse Cefle

**Affiliations:** 1 Division of Rheumatology, Department of Internal Medicine, Kocaeli University Faculty of Medicine, İzmit, Kocaeli 41380 Turkey; 2 Department of Radiology, Kocaeli University Faculty of Medicine, İzmit, Kocaeli Turkey; 3 Department of Internal Medicine, Kocaeli University Faculty of Medicine, İzmit, Kocaeli Turkey

**Keywords:** Computed tomography, Disease progression, Interstitial lung disease, Rheumatoid arthritis

## Abstract

**Introduction:**

Rheumatoid arthritis-associated interstitial lung disease (RA-ILD) presents with variable severity and progression, highlighting the need for effective tools to identify patients at risk. Although CT imaging plays a vital role in the management of RA-ILD, there is a lack of objective methods to predict disease progression. This study investigates the association between semi-quantitative and quantitative CT scoring methods and disease progression in early-stage RA-ILD.

**Methods:**

This observational study analyzed baseline and the first technically evaluable follow-up CT scans of patients who met the 2010 ACR/EULAR classification criteria for RA and were diagnosed with ILD. Only patients with ≤ 5 years between baseline and follow-up scans were included. Semi-quantitative assessments were conducted using the Goh and Warrick scoring systems, while quantitative analyses utilized Vitrea software to measure mean lung attenuation (MLA) and low-, medium-, and high-density lung volumes. Progression risk factors were evaluated using binary logistic regression, with progression defined by changes in CT parameters over time.

**Results:**

A total of 77 RA-ILD patients (45 females, 32 males) were included, with a median follow-up period of 20 months (interquartile range: 7.4–46 months). Disease progression was observed in 34 patients (44.2%). Baseline medium-density volume (MDV), follow-up mean lung attenuation (MLA), and low-density volume (LDV) differed significantly between the progression and non-progression groups (p < 0.05). Quantitative CT parameters demonstrated strong correlations with both the Goh and Warrick scoring systems. Binary logistic regression analysis identified the usual interstitial pneumonia (UIP) pattern on baseline imaging as the only independent predictor of disease progression (odds ratio: 3.1; 95% confidence interval: 1.1–12.4).

**Conclusion:**

In this study of early-stage RA-ILD patients, only the usual interstitial pneumonia (UIP) pattern on baseline HRCT independently predicted disease progression. Neither semi-quantitative scores nor quantitative CT parameters were predictive of progression. However, quantitative CT metrics demonstrated strong correlations with traditional scoring systems, supporting their utility in objectively assessing disease extent.

**Table:** 

**Key Points** • *Quantitative CT analysis offers a standardized and objective tool to evaluate fibrosis extent, supporting early detection and monitoring in RA-ILD management. * • *Quantitative CT parameters demonstrated significant correlations with semi-quantitative scoring methods (Goh and Warrick) in assessing disease extent in RA-ILD.* • *Although semi-quantitative and quantitative methods showed limited ability to predict ILD progression in early-stage RA-ILD, they demonstrate potential for further refinement.*	

## Introduction

Rheumatoid arthritis (RA) is a systemic inflammatory disease primarily affecting the synovial joints and frequently associated with extra-articular manifestations. Among these, interstitial lung disease (ILD) is a common and severe complication that significantly increases the morbidity and mortality burden in RA patients [1, 2].

Clinically significant interstitial lung disease (ILD) is observed in up to 10% of patients with RA, while subclinical interstitial lung abnormalities are even more common [3, 4]. The clinical course of RA-ILD is highly variable; some patients exhibit stable or even improved lung function after diagnosis, whereas others experience a gradual or rapid decline [5]. Several factors have been identified as potential predictors of RA-ILD progression. These include older age, male sex, higher Disease Activity Score in 28 joints (DAS28), the presence of a usual interstitial pneumonia (UIP) pattern, or more extensive ILD findings on high-resolution computed tomography (HRCT). Elevated serum levels of biomarkers such as Krebs von den Lungen- 6 (KL- 6), rheumatoid factor, and anti-cyclic citrullinated peptide (anti-CCP) antibodies have also been implicated [6–9]. Despite the clinical importance of RA-ILD, its natural history and the specific predictors of disease progression remain incompletely understood.

Semi-quantitative evaluation of chest CT remains the gold standard for the radiological assessment of ILD [10]. High-resolution computed tomography (HRCT) is particularly valuable in detecting ILD and identifying disease patterns in patients with rheumatoid arthritis (RA) [11, 12]. While pulmonary function tests (PFTs) and chest X-rays are often used for initial screening, their limited sensitivity can result in missed RA-ILD diagnoses. Many patients with HRCT-confirmed RA-ILD demonstrate normal or near-normal findings on these conventional tests [13]. In contrast, HRCT offers significant advantages by enabling early detection, quantifying the extent of lung involvement, and identifying patterns that reflect disease severity and progression [14]. However, interpreting HRCT images can be challenging due to the complexity of radiographic patterns and the inherent subjectivity of evaluation, which contributes to inter- and intra-observer variability [15] Automated quantification systems hold promise for mitigating this variability by providing consistent, objective assessments of pulmonary abnormalities on HRCT [16–18].

One such quantitative method is quantitative lung densitometry (qLD), which employs computerized algorithms to measure lung parenchymal density in Hounsfield units (HU) on CT scans [19]. By applying predefined HU thresholds, qLD differentiates normal lung tissue from abnormalities associated with conditions such as ILD or emphysema, allowing the calculation of the proportion of lung tissue exhibiting specific pathological features [20]. These advancements underscore the importance of reliable, objective quantification methods to improve the identification of predictors for RA-ILD progression.

In this study, we aimed to evaluate the concordance of a quantitative method, quantitative lung densitometry (qLD), with two widely used semi-quantitative approaches—the Goh scoring system and the Warrick method—for assessing disease progression in RA-ILD patients. Additionally, we explored the utility of these methods in predicting disease progression.

## Methods

We retrospectively analyzed a cohort of RA-ILD patients with follow-up HRCT scans performed between January 2009 and December 2023. All patients fulfilled the 2010 American College of Rheumatology/European League Against Rheumatism (ACR/EULAR) classification criteria for rheumatoid arthritis (RA) [21].

### Data collection

Demographic data, HRCT images, pulmonary function test (PFT) results, and laboratory findings were extracted from electronic medical records. Disease duration was defined as the time from the onset of the first RA symptom to the enrollment date. Baseline disease activity was assessed using the Disease Activity Score in 28 joints–Erythrocyte Sedimentation Rate (DAS28-ESR), calculated within three months of the baseline HRCT scan. All patients underwent a follow-up HRCT scan. Clinical symptoms such as cough, dyspnea, and fatigue, documented during clinic visits at the time of baseline and follow-up HRCT scans, were also obtained. PFT results corresponding to the baseline and follow-up HRCT scans were collected.

#### Definition of progressive lung fibrosis

Progressive lung fibrosis was defined according to the ATS/ERS/JRS/ALAT Clinical Practice Guidelines, requiring at least two of the following three criteria within the previous year: (I) worsening respiratory symptoms, (II) physiological evidence of disease progression (absolute decline in FVC > 5% or DLCO > 10%), or (III) radiological progression [21]. Due to the retrospective design of the study, PFT data were not available for all patients; in such cases, clinical criteria—including newly developed or worsening crackles, dyspnea, or cough—were used to define respiratory symptom progression. When clinical or PFT data were unavailable, radiological progression determined by the radiologist’s assessment was utilized to identify disease progression.

#### Exclusion criteria

Patients with non-fibrotic pulmonary abnormalities (e.g., pneumonia, pulmonary edema, pulmonary thromboembolism, severe left ventricular failure, or significant emphysema) identified via visual HRCT assessment were excluded. When acute abnormalities were suspected (e.g., infection), ILD diagnoses were confirmed by findings on subsequent HRCT scans. Additional exclusion criteria included other respiratory disorders (e.g., asthma, chronic obstructive pulmonary disease), malignancies, or significant pulmonary hypertension, as indicated by clinical or echocardiographic evidence of right heart failure or the need for parenteral therapy. These criteria were established to minimize confounding from non-ILD-related fibrosis on HRCT evaluations.

### Image interpretation

The initial HRCT scan was defined as the imaging obtained at the time of ILD diagnosis, while the follow-up scan was selected from those performed during the first post-diagnosis follow-up evaluation. All patients underwent HRCT using a 64-detector scanner (Aquilion 64; Toshiba Medical Systems, Tokyo, Japan) in a supine position with full inspiration and without contrast enhancement. Scanning parameters included a tube voltage of 120 kV and variable mAs adjusted for patient size using automatic exposure control. Images were acquired with a slice thickness of 1 mm, reconstructed in a 512 × 512 matrix with 1-mm non-overlapping slices using a standard HRCT reconstruction algorithm. Prone scans targeting the lung bases were performed when ground-glass opacities were observed in the posterobasal subpleural regions to distinguish between gravitational opacities and pathological findings. All HRCT images were reviewed with a window level of − 600 Hounsfield units and a width of 1,600 Hounsfield units.

All HRCT scans were re-evaluated by an experienced thoracic radiologist (S.D., with 15 years of experience in thoracic imaging) who was blinded to clinical and laboratory data. ILD diagnosis was based on radiological features, including reticulation, traction bronchiectasis, honeycomb cysts, ground-glass opacities, and airspace consolidation. Pulmonary fibrosis patterns were classified following established guidelines into definite UIP (characterized by honeycombing), probable UIP, nonspecific interstitial pneumonia (NSIP), or organizing pneumonia (OP) using standard radiological terminology [22, 23].

### Semiquantitative assessment of HRCT images

#### Goh scoring

HRCT images were evaluated using the semi-quantitative method described by Goh et al. [24]. Two independent observers (OC and YA), blinded to clinical and lung function data, assessed the scans at five anatomical levels: (1) the origin of the great vessels, (2) the main carina, (3) the pulmonary venous confluence, (4) the midpoint between the third and fifth levels, and (5) just above the right hemidiaphragm. Discrepancies exceeding 20% for continuous scores or more than one grade for categorical scores were resolved through consensus. The HRCT variables analyzed included total disease extent, reticular pattern extent, proportion of ground-glass opacity, and reticular disease coarseness. The global extent of disease was calculated as the average of the extent scores across the five evaluated sections.

#### Warrick scoring

Warrick scoring of the initial and follow-up HRCT images were performed by SD using the semi-quantitative scoring method proposed by Warrick et al. This system identifies five elementary lesions: ground-glass opacities, irregularities in the pleural margins, septal lines, honeycombing, and subpleural cysts. Each lesion is graded on a scale of 1 to 5. These grades contribute to a severity score, calculated as the sum of the individual scores for all observed lesions on HRCT images. The severity score ranges from 0 (no elementary lesions) to 15 (all elementary lesions present). An extent score was subsequently determined for each patient based on the number of lung segments affected by each elementary lesion. Scores were categorized as follows: 1: Lesions present in 1–3 segments; 2: Lesions present in 4–9 segments; 3: Lesions present in more than 9 segments. The total Warrick score was then calculated by summing the severity and extent scores, yielding a range of 0 to 30 (Table 1) [25].

**Table 1:** Criteria used for calculating the Warrick score

Lesions and lung segments		Score
Parenchymal abnormalities		Disease severity score
	Ground-glass opacities	1
	Irregularities in the pleural 2 margins	2
	Septal/subpleural lines	3
	Honeycomb lung	4
	Subpleural cysts	5
Number of lung segments		Disease extent score
	1–3	1
	4–9	2
	> 9	3

### Quantitative assessment of HRCT images

**Fig. 1:**
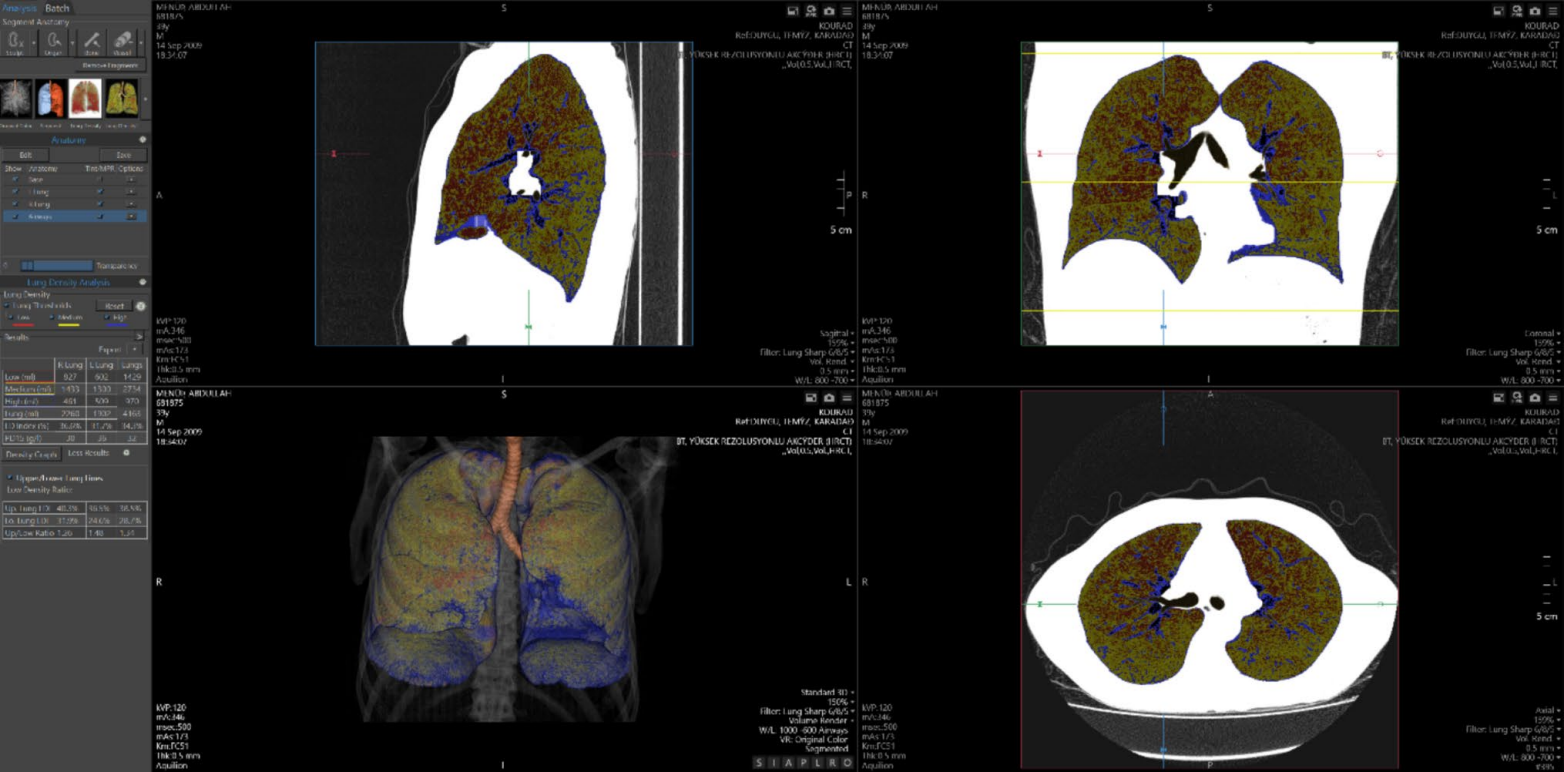
Image quantification analysis with Vitrea® Advanced Visualization software. Reconstruction images of a SSc patient with interstitial lung disease. The relative frequency of attenuation higher than − 720 HU is shown in red, between − 920 to − 720 HU in yellow and − 1020 to − 920 HU in blue

HRCT scans were analyzed using Vitrea® Advanced Visualization software (Canon Group, Minnetonka, MN), a medical imaging and computer-aided diagnostic tool designed for high-precision 3D imaging and volume quantification. One operator (YA), blinded to the patients’ clinical characteristics, performed lung volume and density quantification via the Vitrea® toolbox. Vitrea® supports DICOM-compliant images and provides 3D reconstructions in axial, coronal, and sagittal planes. The software employs advanced segmentation algorithms to differentiate vascular pulmonary structures from normal lung parenchyma and to quantify lung parenchymal volume with high precision. Gaussian smoothing for noise reduction and histogram equalization for contrast enhancement were applied during image preprocessing. All CT scans were saved in DICOM format and subsequently imported into the software for analysis. The system automatically identified and extracted image data, including airways, vessels, and lung parenchyma, to generate 3D reconstructions of the lungs. Segmentation algorithms divided the left and right lungs, and manual corrections were applied to address anatomical variability as needed. Pulmonary vessels were extracted from the segmented lung volumes based on their high density. Using the density mask technique, which highlights voxels within predefined density ranges, the software quantified lung volumes at three density levels: Low-Density Volume (LDV): − 1020 to − 920 HU, associated with emphysema; Medium-Density Volume (MDV): − 920 to − 720 HU, corresponding to functioning lung tissue; High-Density Volume (HDV): − 720 to 0 HU, indicative of pulmonary fibrosis. These density thresholds were automatically adjusted by the software and were consistent with previously reported ranges in the literature [26]. Total lung volume (TLV) was calculated as the sum of LDV, MDV, and HDV, representing the total volume of lung voxels within the range of − 1020 to 0 HU for each patient. This method allowed for detailed quantification of lung parenchymal characteristics, facilitating a comprehensive evaluation of lung involvement in the context of RA-ILD (Fig. 1).

### Statistical analysis

Descriptive statistics for clinical and demographic characteristics were summarized as frequencies and percentages for categorical variables, and as mean ± standard deviation (SD) or median [interquartile range (IQR)] for continuous variables, depending on data distribution. The normality of numerical data was evaluated using visual inspection and the Shapiro–Wilk test. For correlation analysis, Spearman or Pearson correlation analyses were conducted to examine relationships between quantitative lung parameters (low-density volume [LDV], medium-density volume [MDV], high-density volume [HDV], total lung volume [TLV], and mean lung attenuation [MLA]) from baseline HRCT, Goh extent scores, and clinical variables. Clinical parameters included age, DAS28-ESR, rheumatoid factor (RF), anti-CCP titers at RA diagnosis, symptom duration, and forced vital capacity (FVC) at ILD diagnosis. Longitudinal comparison was performed with the Wilcoxon Signed-Ranks Test that was applied to assess changes in quantitative lung parameters and Goh scores over time. Differences in demographic, clinical, laboratory, and radiological data between patients with and without disease progression were analyzed using the Mann–Whitney U test or independent samples t-test for continuous variables, and the Chi-squared test for categorical variables for group comparisons. Multivariable logistic regression analysis was performed to identify independent risk factors associated with ILD progression.

All statistical analyses were conducted using SPSS version 20.0 (IBM Inc., Chicago, IL, USA). Statistical significance was defined as a two-sided p-value < 0.05.

## Results

### Patient demographics and clinical characteristics

**Fig. 2:**
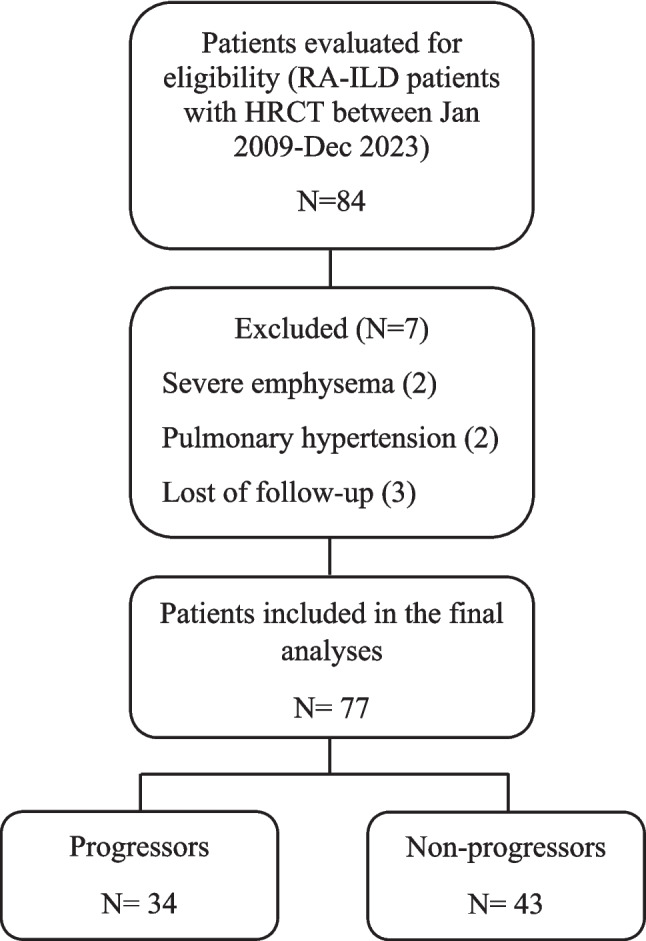
CONSORT-style flow diagram illustrating the patient selection process

A total of 84 RA-ILD patients were screened for eligibility. After applying exclusion criteria—including lack of follow-up HRCT within five years, presence of non-fibrotic pulmonary findings, and comorbid respiratory conditions—77 patients met the inclusion criteria and were enrolled in the final analysis. Among them, 34 patients were classified as progressors and 43 as non-progressors. The patient selection process is summarized in Fig. 2.

The study cohort included 45 women and 32 men, with a mean age of 60.6 ± 8.7 years. The average duration of RA was 8.4 ± 7.1 years, while the mean duration of ILD was 5.4 ± 3.5 years. All demographic and clinical characteristics were recorded at the time of ILD diagnosis (Table 2).

**Table 2:** Comparison of demographic, clinical semi-quantitative, and quantitative scoring methods between progressors and non-progressors groups in patients with RA-ILD

Parameters	All patients (N = 77)	Non-progressors (n = 43)	Progressors (n = 34)	*p*
Gender (Female)	45/77 (58.4%)	26 (60.5%)	19 (55.9%)	0.816
Age at ILD Diagnosis	60.6 ± 8.7	61.3 ± 9.3	59.7 ± 7.9	0.593
Age at RA Diagnosis	54.1 ± 10.7	55 ± 11.5	52.8 ± 9.5	0.527
Duration of RA Symptoms (yrs)	8.4 ± 7.1	8.3 ± 7.5	8.6 ± 6.6	0.724
Baseline RF (titers)	354 ± 710	349 ± 688	361 ± 749	0.786
Baseline Anti-CCP (titers)	214 ± 308	155 ± 207	290 ± 390	0.141
Baseline DAS28-ESR	3.32 ± 1.24	3.57 ± 1.34	3.04 ± 1.08	0.117
Baseline ESR	32.1 ± 25.6	33.6 ± 27.5	30.4 ± 23.6	0.759
Baseline CRP	32.1 ± 25.6	20.6 ± 29.2	11.7 ± 14.5	0.066
Baseline FVC (%)	86.9 ± 16.3	88.8 ± 14.9	83.4 ± 18.8	0.598
Baseline DLCO (%)	64.3 ± 22.6	64.4 ± 24	63.4 ± 19.5	0.949
Smoking (ever)	35/74 (47.3%)	16 (37.2%)	16 (47%)	0.514
Smoking (pack-years)	22.6 ± 15.5	21.8 ± 16	23.4 ± 15.4	0.949
Cough	22/69 (31.9%)	10/36 (27.8%)	12/33 (36.4%)	0.306
Dyspnea	17/69 (24.6%)	10/36 (27.8%)	7/33 (21.2%)	0.363
Crackles	27/72 (37.5%)	14/38 (36.8%)	13/34 (38.2%)	0.852
Interval Between CT Scans (months)	16 [7.5–40]	12.3 [7.4–31]	20 [7.4–46]	0.303

*ILD*, interstitial lung disease; *anti-RF*, anti- Rheumatoid factor; *anti-CCP*, anti-cyclic citrullinated peptide; *DAS28-ESR*, Disease Activity Score- 28; *ESR*, erythrocyte sedimentation rate; *CRP*, C-Reactive Protein; *FVC*, forced vital capacity; *DLCO*, diffusing capacity of carbon monoxide

### Disease progression

Disease progression was observed in 34 of 77 patients (55.9%) based on ATS/ERS/JRS/ALAT Clinical Practice Guidelines. The criteria used for progression categorization were as follows: radiology, PFTs, and clinical findings in 6 patients; radiology and clinical criteria in 17 patients; radiology and PFTs in 8 patients; radiological progression alone (determined by the radiologist) in 3 patients. No significant differences in baseline demographic, disease-related clinical, or laboratory characteristics were observed between progressors and non-progressors (Tables 2 and 3). However, a follow-up UIP pattern, with or without honeycombing, was more frequent among progressors compared to non-progressors (82.4% vs. 53.3%, p = 0.008; and 55.9% vs. 14%, p < 0.001, respectively). Follow-up Warrick severity, extent, and total scores were significantly higher in progressors compared to non-progressors (8.9 ± 3.3 vs. 4.6 ± 2.8; 7.9 ± 2.6 vs. 4.3 ± 2.7; 16.8 ± 5.6 vs. 8.9 ± 5.3, all p < 0.001). Among quantitative parameters, baseline MDV was significantly higher in progressors than non-progressors (16.8 ± 5.6 vs. 8.9 ± 5.3, p < 0.001). Conversely, follow-up MLA and LDV were lower in progressors compared to non-progressors (MLA: 743 ± 108 vs. 792 ± 76, p = 0.011; LDV: 489 ± 445 vs. 753 ± 525, p = 0.017).

**Table 3:** Comparison of semi-quantitative, and quantitative scoring methods between progressors and non-progressors groups in patients with RA-ILD

Parameters	All patients (N = 77)	Non-progressors (n = 43)	Progressors (n = 34)	*p*
Baseline UIP Pattern	47 (61%)	23 (53.5%)	24 (70.6%)	0.127
Baseline NSIP Pattern	13 (16.9%)	7 (16.3%)	6 (17.6%)	0.874
Baseline UIP + Honeycombing	15 (19.5%)	6 (14%)	9 (26.5%)	0.168
Baseline UIP-Honeycombing	32 (41.6%)	17 (39.5%)	15 (44%)	0.685
Follow-up UIP Pattern	51 (66.2%)	23 (53.3%)	28 (82.4%)	0.008
Follow-up NSIP Pattern	12 (15.6%)	7 (16.3%)	5 (14.7%)	0.554
Follow-up UIP + Honeycombing	25 (33.5%)	6 (14%)	19 (55.9%)	< 0.001
Follow-up UIP-Honeycombing	26 (33.8%)	17 (39.5%)	9 (26.5%)	0.229
Baseline Goh Extent (%)	15.4 ± 12.4	16 ± 12.2	14.7 ± 12.9	0.491
Follow-up Goh Extent (%)	20.3 ± 15.5	20.6 ± 15.7	20 ± 15.5	0.980
Baseline Warrick Severity score	5.3 ± 2.9	5 ± 2.7	5.6 ± 3	0.331
Baseline Warrick Extent score	5.1 ± 2.8	4.8 ± 2.6	5.4 ± 2.9	0.548
Baseline Warrick Total score	10.3 ± 5.4	9.7 ± 5.1	11 ± 5.7	0.428
Follow-up Warrick Severity score	6.5 ± 3.7	4.6 ± 2.8	8.9 ± 3.3	< 0.001
Follow-up Warrick Extent score	5.9 ± 3.2	4.3 ± 2.7	7.9 ± 2.6	< 0.001
Follow-up Warrick Total score	12.4 ± 6.9	8.9 ± 5.3	16.8 ± 5.6	< 0.001
Baseline Mean Lung Attenuation (MLA)	754 ± 88	749 ± 90	760 ± 86	0.646
Baseline Low-Density Volume (LDV)	566 ± 488	587 ± 527	540 ± 443	0.917
Baseline Medium-Density Volume (MDV)	1226 ± 458	1046 ± 462	1224 ± 439	0.043
Baseline High-Density Volume (HDV)	522 ± 196	534 ± 214	509 ± 175	0.762
Baseline Total Lung Volume (TLV)	2214 ± 706	2167 ± 718	2273 ± 696	0.517
Follow-up Mean Lung Attenuation (MLA)	771 ± 93	792 ± 76	743 ± 108	0.011
Follow-up Low-Density Volume (LDV)	640 ± 506	753 ± 525	489 ± 445	0.017
Follow-up Medium-Density Volume (MDV)	1029 ± 426	1068 ± 465	975 ± 366	0.453
Follow-up High-Density Volume (HDV)	427 ± 193	395 ± 144	470 ± 240	0.197
Follow-up Total Lung Volume (TLV)	2095 ± 663	2216 ± 700	1933 ± 582	0.101

*UIP*, usual interstitial pneumonia; *NSIP*, non-specific interstitial pneumonia; *MLA*, mean lung attenuation; *LDV*, low-density volume; *MDV*, medium-density volume; *HDV*, high-density volume; *TLV*, total lung volume

### Correlation analysis

We performed a correlation analysis to examine the relationships between quantitative parameters and two visual semi-quantitative scoring methods (Goh and Warrick) for both baseline and follow-up HRCT assessments. In baseline measurements, the Goh extent score correlated with all quantitative parameters to varying degrees, showing the strongest correlations with MLA and HDV (MLA: r = − 0.404, p < 0.001; HDV: r = 0.402, p < 0.001). Other correlations included LDV (r = − 0.262, p = 0.022), MDV (r = − 0.351, p = 0.002), and TLV (r = − 0.275, p = 0.016). Among the Warrick scores, only the extent score showed weak correlations with MLA (r = − 0.252, p = 0.028) and HDV (r = 0.257, p = 0.025).

**Fig. 3:**
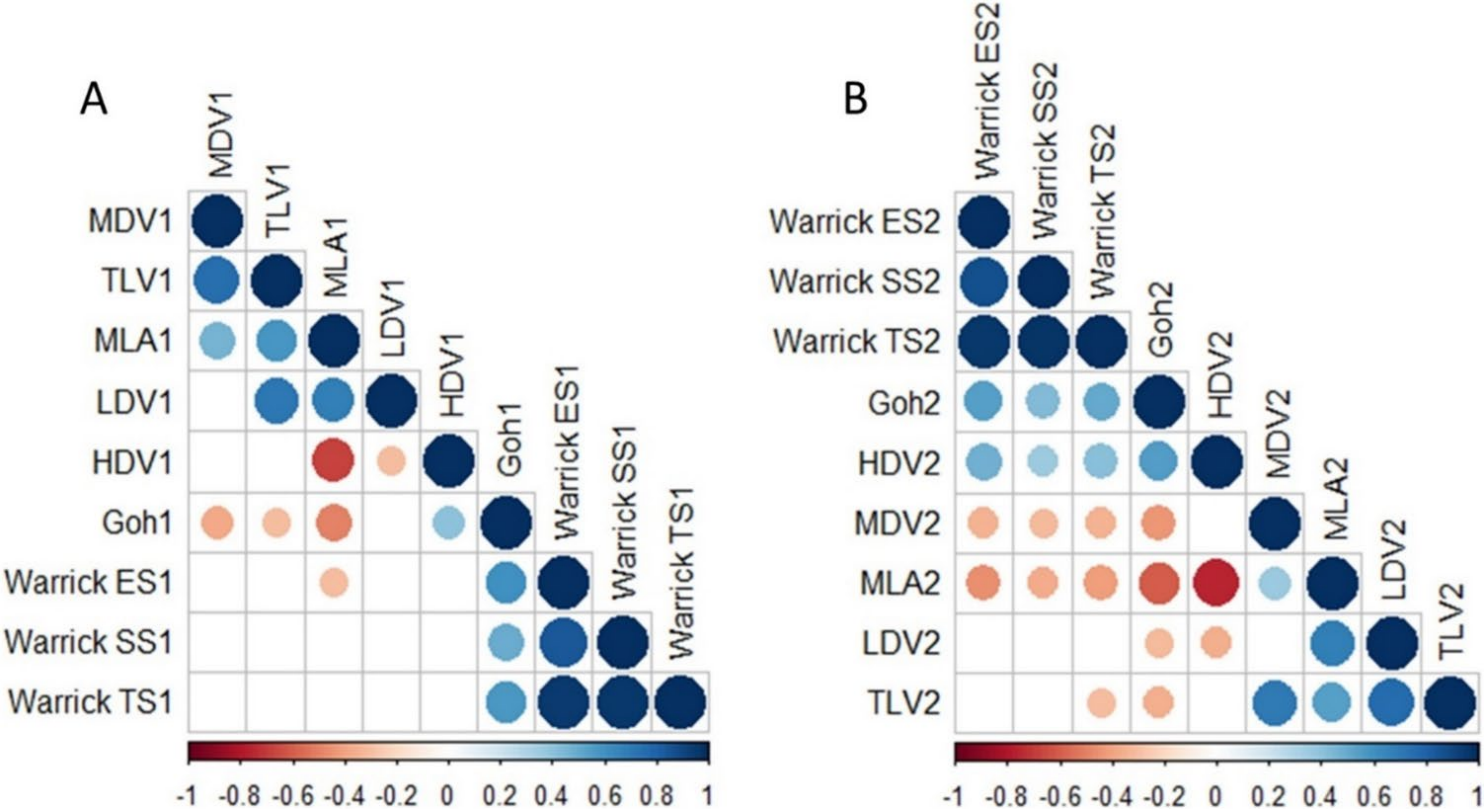
Positive correlations are displayed in blue and negative correlations in red color. Color intensity and the size of the circle are proportional to the correlation coefficients. In the right side of the correlogram, the legend color shows the correlation coefficients and the corresponding colors. MDV1, Baseline Medium-Density Volume; TLV1, Baseline Total Lung Volume; MLA1, Baseline Mean Lung Attenuation; LDV1, Baseline Low-Density Volume; HDV1, Baseline High-Density Volume; Goh1, Baseline Goh Extent; Warrick ES1, Baseline Warrick Extent score; Warrick SS1, Baseline Warrick Severity score; Warrick TS, Baseline Warrick Total score. All the parameters indicated by"2"in the graph represent the"follow-up"parameters

In the follow-up measurements, all visual assessment scores, including the Goh extent score and Warrick severity, extent, and total scores, demonstrated significant correlations with all quantitative parameters. Consistent with baseline results, MLA showed strong correlations with both semi-quantitative methods (e.g., Goh extent score: r = − 0.520; Warrick severity score: r = − 0.428, all p < 0.001). Similarly, HDV exhibited strong correlations with the Goh extent score and Warrick scores (e.g., Goh extent score: r = 0.546; Warrick extent score: r = − 0.479, all p < 0.001). Additionally, HDV was the only quantitative parameter that demonstrated a strong negative correlation with FVC (r = − 0.643, p = 0.043), highlighting its potential relevance in assessing functional decline. Detailed results of the correlation analysis are presented in Table 4 and Fig. 3.

**Table 4:** Correlation between the quantitative parameters and visual assessment methods and pulmonary function tests

***Baseline***	**MLA**		**LDV**		**MDV**		**HDV**		**TLV**	
	*r*	*p*	*r*	*p*	*r*	*p*	*r*	*p*	*r*	*p*
Goh extent score	− 0.404	< 0.001	− 0.262	0.022	− 0.351	0.002	0.402	< 0.001	− 0.275	0.016
Warrick Severity score	− 0.114	0.326	− 0.072	0.538	− 0.184	0.111	0.126	0.276	− 0.133	0.251
Warrick Extent score	− 0.252	0.028	− 0.132	0.256	− 0.190	0.100	0.257	0.025	− 0.161	0.164
Warrick Total score	− 0.176	0.127	− 0.093	0.426	− 0.198	0.086	0.191	0.099	− 0.147	0.204
FVC (%)	0.190	0.375	0.066	0.760	0.214	0.316	− 0.071	0.742	0.228	0.283
DLCO (%)	0.090	0.623	0.214	0.241	0.343	0.055	0.061	0.741	0.229	0.207
***Follow-up***	**MLA**		**LDV**		**MDV**		**HDV**		**TLV**	
	*r*	*p*	*r*	*p*	*r*	*p*	*r*	*p*	*r*	*p*
Goh extent score	− 0.520	< 0.001	− 0.309	0.007	− 0.402	< 0.001	0.546	< 0.001	− 0.339	0.003
Warrick Severity score	− 0.428	< 0.001	− 0.282	0.014	− 0.335	0.003	0.401	< 0.001	− 0.296	0.010
Warrick Extent score	− 0.479	< 0.001	− 0.309	0.007	− 0.350	0.002	0.479	< 0.001	− 0.288	0.012
Warrick Total score	− 0.468	< 0.001	− 0.302	0.008	− 0.368	0.001	0.453	< 0.001	− 0.306	0.008
FVC (%)	0.491	0.150	0.394	0.160	0.200	0.580	− 0.648	0.043	0.200	0.580
DLCO (%)	− 0.109	0.750	− 0.400	0.223	− 0.027	0.937	− 0.309	0.355	− 0.436	0.180

*MLA*, mean lung attenuation; *LDV*, low-density volume; *MDV*, medium-density volume; *HDV*, high-density volume; *TLV*, total lung volume; *FVC*, forced vital capacity; DLCO, diffusing capacity of carbon monoxide

### Prediction of disease progression

A binary logistic regression analysis was performed to identify predictors of ILD progression over a mean follow-up period of two years after the diagnostic HRCT. Variables included gender, age at ILD diagnosis, smoking status, presence of a UIP pattern on initial HRCT, and the quantitative parameters MLA and HDV from baseline HRCT. The UIP pattern on initial HRCT emerged as the only significant predictor of ILD progression, with an odds ratio (OR) of 3.145 (95% confidence interval [CI]: 1.013–9.763, p = 0.047). Neither baseline MLA nor HDV significantly predicted progression, suggesting that radiological patterns, specifically the presence of UIP, play a critical role in identifying patients at higher risk for disease progression in RA-ILD (Table 5).

**Table 5:** The associations of demographic and RA-specific characteristics with ILD progression

	Multivariable model	
	OR (95% CI)	p-value
Gender	0.808 (0.205–3.180)	0.760
Age at ILD diagnosis	0.948 (0.888–1.013)	0.117
Smoking (ever)	0.969 (0.305–3.086)	0.958
UIP patern at baseline	3.145 (1.013–9.763)	0.047
Baseline MLA	1.000 (0.991–1.010)	0.940
Baseline HDV	1.000 (0.996–1.005)	0.848

*ILD*, interstitial lung disease; *UIP*, usual interstitial pneumonia; *MLA*, mean lung attenuation; *HDV*, high-density volume

## Discussion

This study examined the concordance between a quantitative HRCT method and two widely used semi-quantitative methods, the Goh scoring system and the Warrick method in RA-ILD patients. It also evaluated their respective roles in predicting ILD progression. Our findings demonstrated significant correlations between quantitative parameters and the Goh score for baseline HRCT, with specific parameters (MLA and HDV) correlating with the Warrick extent score. On follow-up HRCT scans, all quantitative parameters correlated with scores from both semi-quantitative methods. However, neither initial semi-quantitative nor quantitative parameters predicted ILD progression.

Traditional methods for assessing interstitial lung involvement rely on subjective visual evaluations and semi-quantitative scoring systems [27]. While useful, these approaches are time-consuming and prone to inter- and intra-observer variability [28]. Automated CT quantification systems, extensively used in systemic sclerosis [29, 30], have also shown promise in RA-ILD, allowing objective and reproducible assessment of ILD extent.

One of the aims of our study was to determine the extent to which the parameters identified using quantitative methods correlate with semi-quantitative methods used to assess fibrosis extent. According to our results, we observed a moderate correlation between the quantitative parameters derived from baseline HRCT and the visually determined fibrosis extent using the Goh method. In follow-up HRCTs, we found a stronger correlation between the quantitative parameters and the semi-quantitative scores obtained using both the Goh and Warrick methods [24, 25]. In a study by Oh et al., aimed at determining survival in RA-ILD patients, a quantitative method similar to ours was employed [31]. However, their method differed from ours in that it was based on a pattern/texture approach, rather than the methodology, utilizing HU cut-off/percentile values in the histogram, we applied [32]. Although their study’s objectives and analyzed parameters differed from ours, they reported strong correlations between visually assessed reticulation and QLF, as well as between visually assessed ILD extent and QILD. Humphries et al. demonstrated that baseline data-driven texture analysis (DTA) scores were correlated with baseline FVC (%) and DLCO (%). Furthermore, longitudinal changes in DTA fibrosis scores were associated with changes in FVC and DLCO [33]. Lee et al. extracted computer-generated quantitative scores for ground-glass opacity (QGG), reticular patterns (QLF), and honeycombing (QHC), with the QILD score representing the sum of these patterns from HRCT images. Their results showed correlations between QILD scores and pulmonary function tests, with higher QILD scores associated with lower pulmonary function, particularly for DLCO% [34]. A systematic review, including studies on RA, reported that various quantification methods used in connective tissue diseases showed significant correlations with visual ILD scoring, with R^2^ values ranging from 0.143 (p < 0.01) to 0.687 (p < 0.0001) [35]. These findings demonstrate that fibrosis extent, a key parameter for disease progression that is often not routinely calculated due to time and expertise limitations in daily practice, can be assessed using automated CT quantification methods.

Another aim of our study was to evaluate whether baseline quantitative parameter results could predict progression. In previous studies, the predictive value of quantitative measurements for progression has not been adequately explored; instead, most analyses have focused on predicting survival and mortality. The study by Humphries et al. investigated the relationships between baseline data-driven texture analysis (DTA) scores and RA-ILD severity, survival, and physiological changes over time [33]. Both baseline DTA fibrosis scores and increases in DTA fibrosis on sequential scans were associated with an elevated risk of mortality. Similarly, Lee et al. analyzed the validity of the extent and interval changes in ILD in RA patients using quantitative HRCT imaging methods [34]. Like our study, they explored the potential of the QILD scoring system to characterize both progressive and stable fibrosis but did not assess its role in predicting progression. Oh et al. demonstrated in their study that quantitative lung fibrosis (QLF), quantitative honeycombing (QHC), and quantitative interstitial lung disease (QILD) parameters were associated with 5-year mortality in RA-ILD patients [31]. In our study, we found that baseline quantitative measurements derived from HRCT scans did not predict ILD progression over a mean follow-up period of two years. One potential explanation for this finding could be that the baseline HRCT scans in our cohort captured the early stages of ILD, when fibrosis was minimal, and histogram-based quantitative methods may lack sufficient sensitivity to detect subtle distinctions in these early phases. This may also be supported by the observation that baseline quantitative results showed only weak correlations with the baseline semi-quantitative results from the Goh method. In contrast, follow-up quantitative results exhibited stronger correlations with both semi-quantitative scoring systems. The potential use of texture-based quantification methods for predicting progression remains an area for further investigation, particularly given the need to refine tools for early and reliable assessment of disease trajectory.

One limitation of our study was the retrospective design, which resulted in data loss, particularly for clinical variables based on physical examination. By including baseline and sequential HRCT scans from the early stages of ILD, we aimed to investigate the role of quantitative measurements in predicting progression at the time of ILD diagnosis. However, the retrospective nature of the study restricted the availability of long-term data, as most patients had follow-up HRCT scans obtained within an average of two years, limiting analyses over longer periods. Another limitation was lacking an a priori sample size calculation because of the retrospective design. However, a post hoc power analysis based on the observed effect size for the association between baseline UIP pattern and disease progression (Cohen’s h = 0.397) indicated a statistical power of approximately 69.2%. While this suggests moderate power to detect stronger associations, the study may have been underpowered to identify more subtle predictive effects of semi-quantitative and quantitative imaging parameters.

## Conclusion

Our study revealed that the Goh and Warrick scoring systems, as well as quantitative analyses, were not predictive of progression. The integration of quantitative imaging techniques into routine practice could enhance early detection and monitoring of RA-ILD, addressing challenges related to time and radiologist expertise. These methods offer the potential for individualized patient monitoring, improving disease management, and enabling tailored therapeutic strategies. Future research should focus on refining quantification methods to predict disease progression reliably, particularly in the early stages of ILD.

## Data Availability

The datasets used and/or analysed during the current study are available from the corresponding author on reasonable request.
